# Bifunctional Phenylalanine/Tyrosine Ammonia-Lyase (PTAL) Enhances Lignin Biosynthesis: Implications in Carbon Fixation in Plants by Genetic Engineering

**DOI:** 10.3390/biology13090742

**Published:** 2024-09-22

**Authors:** Ye Yuan, Chao-Lei Sheng, Li-Hao Pang, Bao-Rong Lu

**Affiliations:** Ministry of Education Key Laboratory for Biodiversity Science and Ecological Engineering, Department of Ecology and Evolutionary Biology, Fudan University, Songhu Road 2005, Shanghai 200438, China; yuany19@fudan.edu.cn (Y.Y.); 18210700001@fudan.edu.cn (C.-L.S.); 20110700002@fudan.edu.cn (L.-H.P.)

**Keywords:** PTAL, lignin biosynthesis, Arabidopsis, weedy rice, phenylalanine, tyrosine, bioengineering, carbon fixation

## Abstract

**Simple Summary:**

Phenylalanine/tyrosine ammonia-lyase (PTAL) is a bifunctional enzyme that can deaminate both phenylalanine and tyrosine. To explore the efficiency of lignin biosynthesis with the bifunctional enzyme, we applied a comparable experimental system consisting of transgenic and non-transgenic Arabidopsis and crop-weedy rice hybrid (CWRH) lineages to analyze lignin-biosynthesis associated metabolites. We found substantial increases in *EPSPS* gene expression, phenylalanine and tyrosine contents, total lignin and its monolignols (lignin units), and biomass in the experimental materials. Importantly, these results demonstrated that overexpressing the *EPSPS* genes in Arabidopsis and CWRH lineages increased phenylalanine and tyrosine levels, which significantly promoted lignin biosynthesis and biomass production in the transgenic CWRH lineages serve as a sink for plant carbon fixation. These findings are important for a better understanding of the PAL and PTAL’s functions in different plant species, in addition to efficient carbon fixation by genetic engineering in plants.

**Abstract:**

Lignin is a key metabolite for terrestrial plants. Two types of aromatic amino acids, phenylalanine (Phe) and tyrosine (Tyr), serve as the precursors for lignin biosynthesis. In most plant species, Phe is deaminated by Phe ammonia-lyase (PAL) to initiate lignin biosynthesis, but in grass species, Phe and Tyr are deaminated by Phe/Tyr ammonia-lyase (PTAL). To understand the efficiency of PAL and PTAL, we used transgenic and non-transgenic Arabidopsis with PAL and crop-weedy rice hybrids (CWRH) with PTAL to analyze lignin-biosynthesis-associated metabolites. The transgenic plants overexpressed the exogenous 5-enolpyruvylshikimate-3-phosphate synthase (*EPSPS*) gene, whereas the non-transgenic plants normally expressed the endogenous *EPSPS* gene. Our results show significantly increased Phe/Tyr contents in transgenic Arabidopsis and CWRH plants, leading to substantially increased lignin and biomass. In addition, the PTAL pathway promotes a much greater proportion of increased lignin and biomass in transgenic CWRH than in transgenic Arabidopsis lineages. Evidently, more efficient lignin biosynthesis characterized the grass species possessing the PTAL pathway. These findings are important for a better understanding of the PAL and PTAL’s functions in the phenylpropanoid metabolic pathways in the evolution of plant species. These findings also have great value for implications such as effective carbon fixation by enhancing lignin biosynthesis through genetic engineering of their key genes in appropriately selected plant species.

## 1. Introduction

Lignin—a complex phenolic polymer of multiple carbon plays a key role in plants colonizing the terrestrial ecosystems from the ocean in early times when plants initially faced tremendous stresses [1,2,3]. As an important secondary metabolite for the growth and development of plants, lignin evolved to meet the challenge of ultraviolet radiation, desiccation, and the coevolved herbivores and pathogens [1,2,3]. Undoubtedly, the biosynthesis of lignin and other phenolic metabolic compounds allows plants to adapt to terrestrial ecosystems with tremendous differences in environmental conditions, bringing great diversity and flourishment to the terrestrial plants [4]. Lignin enhances the strength of plant cell walls and hydrophobic properties, promotes mineral transportation through the vascular bundles, and protects plants against insect pests and pathogens as an important barrier [5,6,7]. Besides, lignin has many biological functions for the growth and development of plants, such as inhibiting plant deformity development through the transcriptional procedures and signaling pathways and prompting plant lodging resistance and environmental stress adaptation [3,8]. Therefore, generating knowledge on lignin’s complex compounds and biosynthesis will be essential for better understanding biological processes and establishing their solid theoretical foundations. Such knowledge will be a valuable guide for manipulating lignin contents and their associated compounds, such as biomass, through genetic engineering in plant breeding for agricultural, food, and biofuel productions.

**Figure 1:**
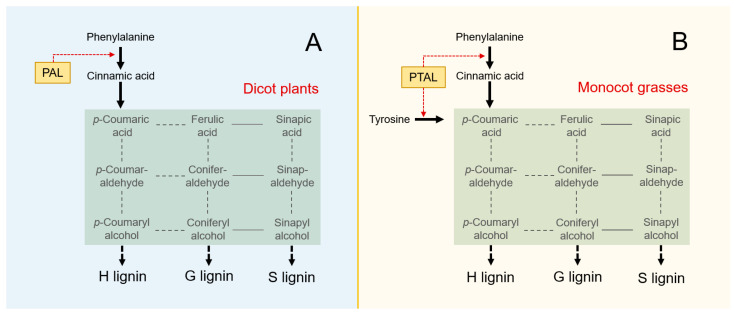
The model for lignin biosynthesis in dicot plants (**A**) and monocot grasses (**B**). Most dicot plant species only possess the monofunctional phenylalanine (Phe) ammonia-lyase (PAL) to deaminate Phe during the initial lignin biosynthesis stages (**A**). However, the monocot grasses include the bifunctional phenylalanine/tyrosine (Tyr) ammonia-lyase (PTAL) to deaminate both Phe and Tyr in lignin biosynthesis (**B**) [11]. The primitive monofunctional PAL pathway possesses a single channel to use Phe (dashed red arrow in (**A**)), whereas the evolved bifunctional PTAL pathways possess two channels to use both Phe and Tyr (dashed red arrows in (**B**)). Shaded squares indicate the common pathways of lignin biosynthesis both in dicots and grasses. Black dashed lines indicate multiple intermediate reactions [13].

There are two types of aromatic amino acids produced in the shikimate pathway, namely phenylalanine (Phe) and tyrosine (Tyr), that serve as the key precursors for lignin biosynthesis [9,10]. Commonly, the first step for lignin biosynthesis is to deaminate Phe into *trans*-cinnamic acid by the monofunctional phenylalanine ammonia-lyase (PAL) followed by the subsequential syntheses (see Figure 1A). However, it has been reported recently in the grass species that the lignin biosynthesis can be accomplished by the bifunctional phenylalanine/tyrosine ammonia-lyases (PTAL), which can deaminate both Phe and Tyr (see Figure 1B), even though PAL remains its original function in the grasses [11,12]. Although detailed knowledge concerning the above phenomenon is still limited, understanding the efficiency of the PAL and PTAL systems or pathways in terms of lignin biosynthesis is very important. With the presence of PTAL, the phenylpropanoid metabolism, particularly the lignin biosynthesis, prefers the S-lignin and wall-bound coumarate biosynthesis [10]. However, in grass species that already have the bifunctional PTAL system, the mechanisms for enhanced lignin biosynthesis and increases in total lignin content still lack reliable evidence. In addition, detailed information concerning how the PTAL system contributes to efficient lignin biosynthesis remains unknown.

Lignin constitutes up to ~30% of dry matter or biomass in plants [14]. Together with cellulose, these constitute the most abundant renewable organic polymers on the Earth [14]. Theoretically, with the increase in Phe and Tyr levels through genetic engineering of the targeted genes in the relevant pathways, the biomass of plants can be considerably increased. This is because Phe and Tyr produced from the shikimate pathway are the key precursors for lignin biosynthesis [9,15]. In return, the increased biomass by plants will contribute more to the available resources for fuel and feedstock chemicals [16,17]. More importantly, plant biomass acts as a carbon sink that plays an important role in carbon fixation and neutrality [18]. Whether regulating the function of PTAL in plants can contribute to effective carbon fixation will provide insight into future implications in carbon neutrality through genetic engineering technologies. Such knowledge can be very useful in selecting the appropriate types of plants (e.g., with PAL or PTAL) for genetic engineering to achieve the above-mentioned goals more efficiently.

Previous studies indicated that the contents of tryptophan (Trp), Phe, and Tyr could increase substantially in transgenic Arabidopsis and crop-weedy rice hybrid (CWRH) lineages overexpressing the 5-enolpyruvoylshikimate-3-phosphate synthase (*EPSPS*) gene, in comparison with their non-transgenic counterparts normally expressing the *EPSPS* gene [19,20,21]. Those findings suggest that engineering of the *EPSPS* gene in the shikimate pathway can regulate the production of growth- and development-associated metabolites, such as Trp, Phe, and Tyr, in plants. Studies also indicated that overexpressing *EPSPS* gene could enhance some physiological and phenotypical traits, such as auxin content, biomass, and photosynthesis [19,20,21,22,23]. The downstream metabolites of EPSPS, including Trp, Phe, and Tyr, exhibited considerable changes in contents at different *EPSPS* expression levels. Accordingly, to establish an experimental system with plant lineages that express the *EPSPS* gene normally or exceedingly through genetic engineering. Such an experimental system will facilitate the evaluation of lignin biosynthesis efficiency in dicot plants (possessing PAL) and grass plants (possessing PTAL) by comparing the lineages with low or high Phe and Tyr levels. Accurate analyses of the proportion of increase in Phe, Tyr, lignin, and biomass content between plant lineages with low or high levels of target metabolites can answer our questions concerning the efficiency of monofunctional PAL or bifunctional PTAL in lignin biosynthesis. 

**Figure 2:**
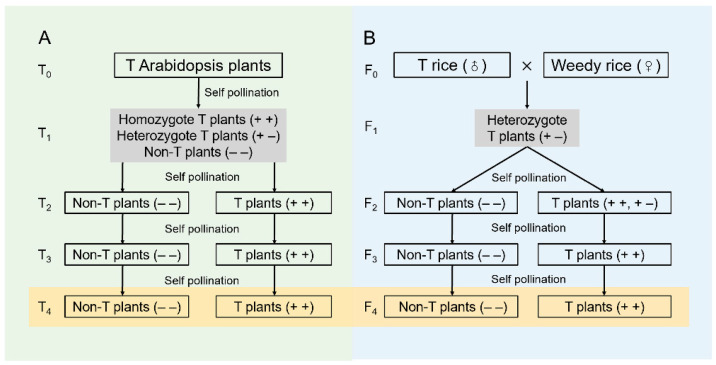
An illustration showing the production of transgenic and non-transgenic Arabidopsis lineages (**A**) and crop-weedy rice hybrid (CWRH) lineages (**B**) and the pedigrees of their derived transgenic and non-transgenic lineages through successive self-pollination and transgene identification [19,20]. + + and + − indicate homozygous and heterozygous transgenic (T) plants, respectively; − − indicates homozygous non-transgenic (Non-T) plants. Only T_4_ Arabidopsis and F_4_ CWRH lineages (orange-colored background) were used in this study.

In this study, we used the dicot (Arabidopsis) and grass (rice) plants to establish an experimental system with the T_4_ and F_4_ homozygous non-transgenic and transgenic plant lineages through genetic engineering of the *EPSPS* gene (Figure 2) to achieve the differential *EPSPS* expression levels. The primary objectives of the study are to compare (i) the content of Phe and Tyr in non-transgenic and transgenic dicot and grass lineages; (ii) the efficiency of lignin biosynthesis in dicot with PAL and grass species with PTAL by determining the increased content and ratio of total lignin and its monolignols; and (iii) the differences and increased ratios in biomass between the non-transgenic and transgenic dicot and grass lineages. Knowledge generated from this study will improve our understanding of the efficiency of lignin biosynthesis between dicots (with monofunctional PAL) and grasses (with bifunctional PTAL). It will also facilitate our efficient strategies for selecting appropriate plant candidates used for future genetic engineering, which aims to enhance the desired bioproducts. 

## 2. Materials and Methods

### 2.1. Plant Materials

To determine the efficiency of lignin biosynthesis in dicots possessing the PAL pathway, we established the T_4_ homozygous transgenic Arabidopsis (*Arabidopsis thaliana*, Columbia 0) lineages overexpressing the exogenous *EPSPS* genes and their homozygous non-transgenic counterparts normally expressing the endogenous *EPSPS* gene (Figure 2A), following the method of Fang et al. [20]. In addition, to better understand the efficiency of lignin biosynthesis in grasses possessing the PTAL pathway, we produced the F_4_ homozygous transgenic crop-weedy rice (*Oryza sativa*) hybrid (CWRH) lineages overexpressing the *EPSPS* genes and their homozygous non-transgenic counterparts normally expressing the endogenous *EPSPS* gene (Figure 2B), following the method of Wang et al. [19]. The dicot Arabidopsis lineages (having PAL) were used to compare to the CWRH lineages (having PTAL) in this study. Both transgenic Arabidopsis and CWRH lineages contained the same *EPSPS* transgenes transformed from cultivated rice [19,20].

To ensure the fairness of the parallel comparison, only the T_4_ (for Arabidopsis) and F_4_ (for CWRH) lineages were included in the analyses in this study (highlighted by orange color in Figure 2). The homozygous transgenic (+ +) and non-transgenic (− −) T_4_ and F_4_ lineages were produced and randomly selected from the T_1_ and F_1_ transgenic plants through consecutive self-pollination and transgene identification in the segregated populations (Figure 2). The methods to identify and select the transgenic and non-transgenic plants followed the description by Fang et al. and Wang et al. [19,20]. The DNA sequences of the applied primer pairs for identifying the transgenic Arabidopsis plants were 5′-acgaatgagggagagaccga-3′ (forward) and 5′-accatcagcgaagagtgcaa-3′ (reverse); the primer pairs for identifying the transgenic CWRH plants were 5′-gtggcttcctggagagtaaag-3′ (forward) and 5′-gttcctgacgaaagtgcttaga-3′ (reverse). Consequently, four T_4_ Arabidopsis lineages, including two transgenic and two non-transgenic lineages, as well as four F_4_ CWRH lineages, including two transgenic and two non-transgenic lineages, were included in the study, respectively.

### 2.2. Measurement of Gene Expression in Different Lineages

Plants from the two non-transgenic and two transgenic T_4_ Arabidopsis lineages (40 days after seed gemination, DAG), as well as those from the two non-transgenic and two transgenic F_4_ CWRH lineages (45 DAG) were included to measure the expression levels of *EPSPS*.

To confirm the expression of the *EPSPS* genes in transgenic Arabidopsis and CWRH lineages, a total of 30 randomly selected plants from a lineage divided into five groups (*n* = 5 replicates), each containing six plants, were included for analyses. Six plants from each replicate were mixed and subjected to the analyses based on the real-time quantitative PCR (RT-*q*PCR). The primer pairs for each selected gene were designed by Primer Premier 6.0 and were confirmed specific to the gene by NCBI Primer-BLAST (https://www.ncbi.nlm.nih.gov/tools/primer-blast/, accessed on 2 September 2024). The primer sequences for *AtEPSPS* were 5′-aaggatgcgaaagagg-3′ (forward) and 5′-caacccgacaaccaa-3′ (reverse); for *OsEPSPS* were 5′-gcagttggaccatcagcgaag-3′ (forward) and 5′-ctgttgagaaggatgcgaaaga-3′ (reverse). The RT-*q*PCR reactions were performed on ABI 7300 with Takara TB Green^®^ *Premix Ex Taq*™ II (RR820A) (TaKaRa, Beijing, China). The reaction volume was 25.0 μL, containing 12.5 μL TB Green *Premix Ex Taq* II (Tli RNaseH Plus), 2.0 μL cDNA, 1.0 μL forward primer and 1.0 μL reverse primer (10 μM), and 8.5 μL ddH_2_O, following the manufactory protocols. The RT-*q*PCR procedure was determined as 95 °C for 30 s and 40 cycles of 95 °C for 5 s, 60 °C for 30 s, and 72 °C for 30 s. The relative gene expression level was calculated with the 2^−△△Ct^ method [24] using *RPS27AC* (UBQ5) and *ACT1* (ACTIN-1) as the reference genes in Arabidopsis and rice, respectively. The sequences of primer pair for *RPS27AC* were 5′-aatgtgaaggcgaagatccaagac-3′ (forward) and 5′-agacggaggacgagatgaagc-3′ (reverse); for *ACT1* were 5′-tccatcttggcatctctcag-3′ (forward) and 5′-gtaccctcatcaggcatctg-3′ (reverse). Hence, a total of 240 plants were included in the RT-*q*PCR analyses.

### 2.3. Measurement of Phenylalanine and Tyrosine in Different Lineages

Plants from the two non-transgenic and two transgenic T_4_ Arabidopsis lineages, and those from the two non-transgenic and two transgenic F_4_ CWRH lineages were included to measure the phenylalanine (Phe) and tyrosine (Tyr) contents and biomass.

For the purpose of measuring the contents of Phe and Tyr, a total of 30 randomly selected plants from a lineage being divided into five groups (*n* = 5 replicates), each containing six plants, were included for analyses. Six plants from each replicate were mixed and subjected to the analyses based on liquid chromatography–mass spectrometry (LC-MS) [25]. A total of 240 plants were included in the LC-MS analyses.

### 2.4. Measurement of Biomass, Total Lignin, and Monolignols in Different Lineages

Fresh weight and dry weight were measured to estimate the biomass between the non-transgenic and transgenic lineages. A total of 30 randomly selected plants from each of the relevant T4 Arabidopsis lineages and the F_4_ CWRH lineages were included for analyses, respectively. The 30 plants from each lineage were also divided into five groups (*n* = 5 replicates), each containing six plants. The above-ground portion of the Arabidopsis (40 DAG) and CWRH (45 DAG) plants were harvested. The fresh weight of each plant was immediately measured after harvesting. All the measured plants were then placed in an oven at 105 °C for ~1 h to dry. The dry weight was measured using the dried plants to the constant weight in an oven at 60 °C. Accordingly, a total of 240 plants were used for the determination of biomass.

Plants from the two non-transgenic and two transgenic T4 Arabidopsis lineages and those from the two non-transgenic and two transgenic F_4_ CWRH lineages were included to measure the content of total lignin and three major monolignols, namely *p*-coumaryl alcohol (H), coniferyl alcohol (G), and sinapyl alcohol (S). A total of 30 randomly selected plants from each lineage were divided into five groups (*n* = 5 replicates), each containing six plants. Accordingly, a total of 240 plants were included to measure the contents of the total lignin and monolignols. The above-ground portion of the Arabidopsis and CWRH plants were harvested 40 and 45 DAG, respectively, and dried to constant weight. The dry plants were ground into powders using a ball mill at the frequency of 28 Hz for 2 min. The total lignin was extracted and then measured using the Klason method and ultraviolet spectrophotometry following the description of Song et al. [26]. The contents of the three monolignols were measured using the thioacidolysis gas combined with chromatography-mass spectrometry (GC-MS) following the description of Song et al. [26].

### 2.5. Statistical Analysis

Statistical analysis employed a two-tailed, independent-sample Student’s *t*-test to identify significant differences between non-transgenic and transgenic Arabidopsis and CWRH lineages. The Shapiro–Wilk test was carried out to confirm the normal distribution of the included data before the Levene’s test was conducted to assess the homogeneity of variances. All statistical analyses were performed using Microsoft Excel 2021. Diagrams were designed in Microsoft PowerPoints 2021. Data visualizations were performed using GraphPad Prism 8.0.1.

## 3. Results

### 3.1. Expression of the EPSPS Gene in Non-Transgenic and Transgenic Lineages

**Figure 3:**
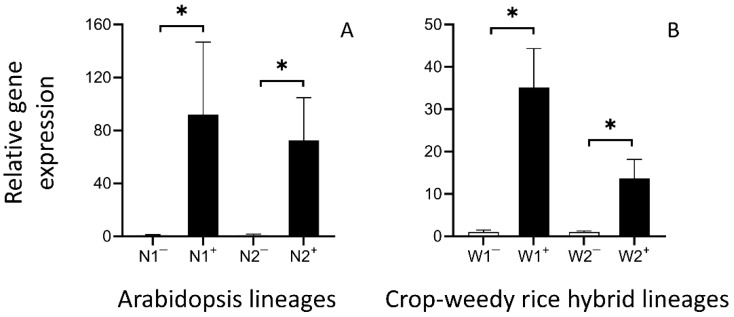
Expression levels of the *EPSPS* genes in the transgenic lineages (N1^+^ and N2^+^, W1^+^ and W2^+^, black columns) and their corresponding non-transgenic lineages (N1^−^ and N2^−^, W1^−^ and W2^−^, white columns). (**A**): Arabidopsis lineages; (**B**): crop-weedy rice hybrid lineages. Error bars represent standard errors (SE). * *p* < 0.05.

Our results showed significant increases (*p* < 0.05) in the expression level of the *EPSPS* gene in the transgenic plants, both in dicot Arabidopsis and monocot CWRH (crop-weedy rice hybrid) lineages. The expression level of the *EPSPS* genes was about 70–100 folds higher in the transgenic Arabidopsis lineages than in their corresponding non-transgenic counterparts (Figure 3A). Similarly, the expression level of the *EPSPS* genes was about 10–40 folds higher in the transgenic CWRH lineages than in their corresponding non-transgenic counterparts (Figure 3B). These results confirmed that the exogenous *EPSPS* transgene both in transgenic Arabidopsis lineages and in transgenic CWRH lineages significantly promoted the *EPSPS* expression levels.

### 3.2. Contents of Phenylalanine and Tyrosine in Non-Transgenic and Transgenic Lineages

**Figure 4:**
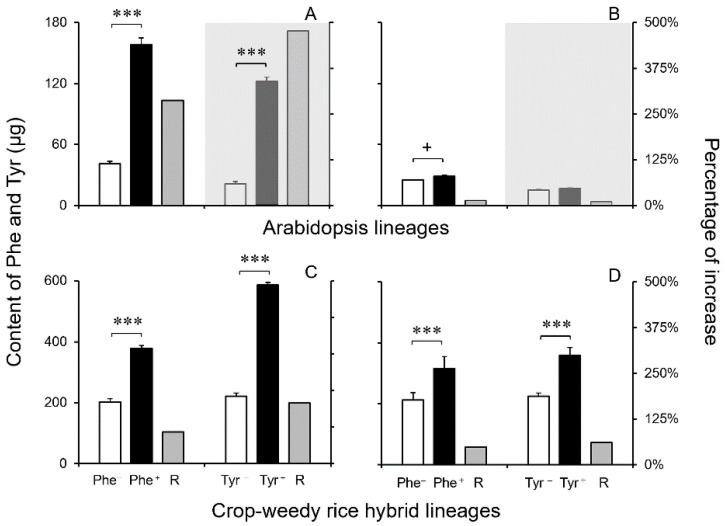
Contents of phenylalanine (Phe) and tyrosine (Tyr) in the *EPSPS* transgenic lineages (Phe^+^ and Tyr^+^, black columns) and their corresponding non-transgenic lineages (Phe^−^ and Tyr^−^, white columns), in addition to the ratios (R) of increased Phe and Tyr (grey columns). (**A**,**B**): Arabidopsis lineages; (**C**,**D**): crop-weedy rice hybrid (CWRH) lineages. Shaded parts indicate Tyr generated from the shikimate pathway, not being used in the lignin biosynthesis in Arabidopsis that only possesses the monofunctional Phe ammonia-lyase (PAL). Error bars represent standard errors (SE). + *p* < 0.1 (close to significance); *** *p* < 0.001.

In general, the measured contents of phenylalanine (Phe) and tyrosine (Tyr) increased significantly in the transgenic lineages overexpressing the *EPSPS* gene in the dicot Arabidopsis and monocot CWRH lineages, although with considerable variation. This observation is based on the comparison of the liquid chromatography-mass spectrometry (LC-MS) examination between the transgenic lineages and their corresponding non-transgenic counterparts that only expressed the *EPSPS* gene normally (Figure 4A–D). The left y-axes represent the values of the content (white and black columns), and the right y-axes represent the percentage increase of the transgenic lineages compared to their non-transgenic counterparts (grey columns) (Figure 4A–D).

For the 40-day-old Arabidopsis plants (*n* = 5 × 6), the average Phe and Tyr contents increased by 13.5~287.7% and 10.4~477.7%, respectively, in the two measured transgenic lineages, compared with those in their corresponding non-transgenic counterparts (Figure 4A,B). These results showed significant (*p* < 0.001) increases or close-to-significant (*p* < 0.1) increases (Figure 4A,B) in the Phe contents in the two transgenic Arabidopsis lineages. However, the significant increases (*p* < 0.001) in Tyr contents were only observed in one of the transgenic Arabidopsis lineages (Figure 4A,B), which belonged to two independent transgenic events. 

For the 45-day-old transgenic CWRH plants (*n* = 5 × 6), the average Phe and Tyr contents increased by 48.6~87.1% and 60.4~165.3%, respectively, in the two measured transgenic lineages, compared with those in their corresponding non-transgenic counterparts (Figure 4C,D). The Phe and Tyr contents increased significantly (*p* < 0.001) in the two measured transgenic CWRH lineages (Figure 4C,D). Noticeably, the increased ratios of Tyr contents were somehow greater than those of Phe contents in the transgenic CWRH lineages. Altogether, our results indicated that overexpression of the *EPSPS* gene substantially enhanced the production of Phe and Tyr. Noticeably, Phe and Tyr were synthesized in the shikimate pathway as downstream products of EPSPS in both dicot plants and monocot grasses.

### 3.3. Total Lignin, Monolignols, and Biomass in Non-Transgenic and Transgenic Lineages

**Figure 5:**
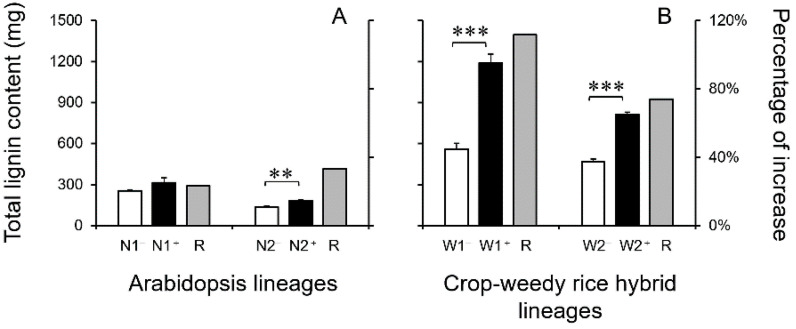
The content of total lignin and the ratio (R) of increased total lignin (grey columns) in the transgenic lineages (N1^+^ and N2^+^, W1^+^ and W2^+^, black columns) and their corresponding non-transgenic counterparts (N1^−^ and N2^−^, W1^−^ and W2^−^, white columns). (**A**): Arabidopsis lineages; (**B**): crop-weedy rice hybrid (CWRH) lineages. Error bars represent standard errors (SE). ** *p* < 0.01; *** *p* < 0.001.

***Content of total lignin*:** The increased proportion of lignin contents in transgenic lineages overexpressing the *EPSPS* (trans)gene and in their corresponding non-transgenic counterparts normally expressing the *EPSPS* gene could indicate the efficiency of lignin biosynthesis in plants. As expected, results from this study indicated only some degrees of increase in the total lignin content in the transgenic Arabidopsis lineages, with the dicot plants only possessing the monofunctional PAL pathway (Figure 5A). In contrast, a significantly increased proportion (*p* < 0.001) of the total lignin content was detected in the transgenic CWRH lineages, the grass plants possessing the bifunctional PTAL pathway (Figure 5B). These results suggested that the transgenic grass (CWRH) plants with the bifunctional PTAL pathway were much more efficient in lignin biosynthesis than the transgenic dicot Arabidopsis plants, which only had the monofunctional PAL pathway. 

For the 40-day-old Arabidopsis plants (*n* = 5 × 6), the increased ratios of the total lignin content were about 23.4~33.4% in the transgenic lineages, compared with those in their corresponding non-transgenic counterparts (Figure 5A). Noticeably, there was only one transgenic Arabidopsis lineage (N2) that showed a significant increase in the total lignin content. However, for the 45-day-old CWRH plants (*n* = 5 × 6), the increased ratios of the total lignin content were about 74.0~111.6% (*p* < 0.001) in the transgenic grass lineages, compared with those in their corresponding non-transgenic counterparts (Figure 5B). These obtained results suggested that the biosynthesis of the total lignin was substantially more efficient in the transgenic grass (CWRH) plants than in the transgenic dicot Arabidopsis plants, regardless of the increased ratios of Phe and Tyr in these plants. This finding proved that lignin biosynthesis was much more efficient in the grass plants with the bifunctional PTAL pathway that could deaminate both Phe and Tyr than in the dicot Arabidopsis plants with monofunctional PAL pathway that could only deaminate Phe.

**Figure 6:**
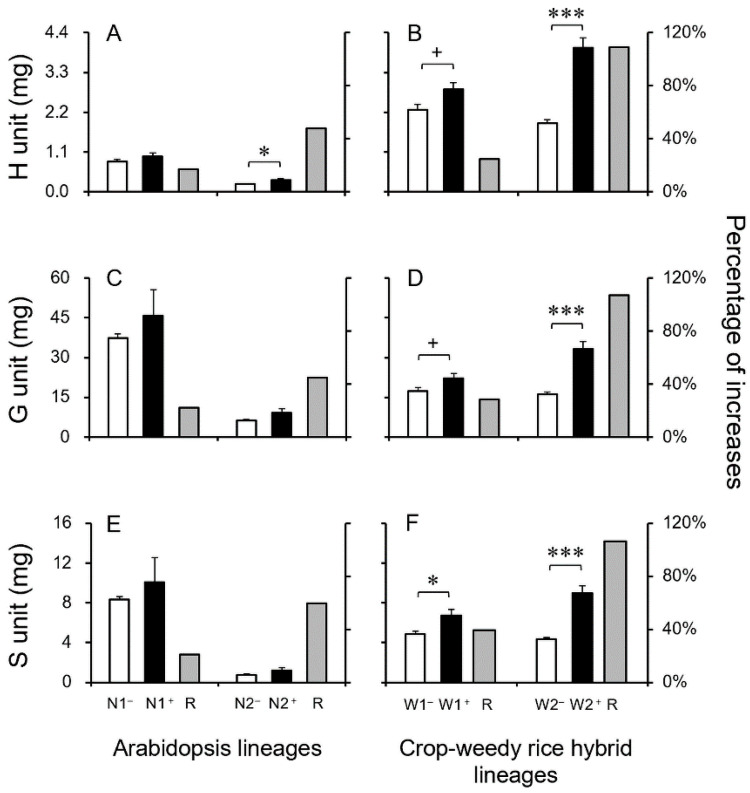
The content of the H-, G-, and S-lignin units and the ratio (R) of increased lignin units (grey columns) of the transgenic lineages (black columns) in Arabidopsis (N1^+^ and N2^+^) and crop-weedy rice hybrid (CWRH) lineages (W1^+^ and W2^+^) and their corresponding non-transgenic counterparts (N1^−^ and N2^−^, W1^−^ and W2^−^, white columns). (**A**,**C**,**E**): Arabidopsis lineages; (**B**,**D**,**F**): CWRH lineages. Error bars represent standard errors (SE). + *p* < 0.1 (close to significance); * *p* < 0.05; *** *p* < 0.001.

***Content of Monolignols*:** The authors further measured the contents of the three lignin monolignols using the GC-MS analysis to determine whether lignin biosynthesis was more efficient in the grass CWRH plants with the bifunctional PTAL pathway. Generally, the obtained results indicated significant increases in the H-, G-, and S-lignin contents in the transgenic grass (CWRH) lineages (Figure 6B,D,F), all of which had substantially increased Phe and Tyr. In contrast, the observed increases in the H-, G-, and S-lignin contents were not so outstanding in the transgenic dicot Arabidopsis lineages, in which only one transgenic lineage (N2) showed a significant increase in the H-lignin content (Figure 6A,C,E).

For the 40-day-old Arabidopsis plants (*n* = 5 × 6), the increased ratios of H-, G-, and S-lignin were only about 17.1~47.7%, 22.3~45.0%, and 20.9~59.7%, respectively, compared with those in their corresponding non-transgenic counterparts (Figure 6A,C,E). However, for the 45-day-old CWRH plants (*n* = 5 × 6), the increased ratios of the H-, G-, and S-lignin contents were about 24.7~109.0%, 28.4~107.0%, and 39.3~106.4%, respectively, in the transgenic CWRH lineages, compared with those in their corresponding non-transgenic counterparts (Figure 6B,D,F). Noticeably, the trend of increases in the H-, G-, and S-lignin content was consistent with that in the total lignin content, both in transgenic Arabidopsis and CWRH lineages. Altogether, these results demonstrated that overexpressing the *EPSPS* gene substantially increased the H-, G-, and S-lignin production in the grass (CWRH) plants with the bifunctional PTAL pathway through more efficient lignin biosynthesis.

**Figure 7:**
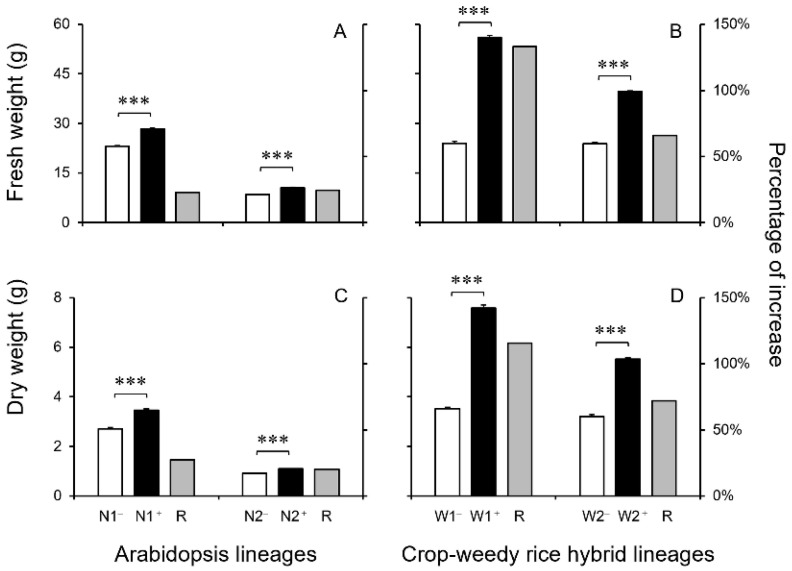
Fresh weight (**A**,**B**) and dry weight (**C**,**D**) in the *EPSPS* transgenic lineages (N1^+^ and N2^+^, W1^+^ and W2^+^, black columns) and their corresponding non-transgenic counterparts (N1^−^ and N2^−^, W1^−^ and W2^−^, white columns), in addition to the ratio (R) of increased fresh and dry weight (grey columns). (**A**,**C**): Arabidopsis lineages; (**B**,**D**): crop-weedy rice hybrid (CWRH) lineages. Error bars represent standard errors (SE). *** *p* < 0.001.

***Content of biomass*:** The content of biomass, as presented either by fresh weight or dry weight in different lineages, could be considerably influenced by lignin, whose precursors were aromatic amino acids, including phenylalanine (Phe) and tyrosine (Tyr). Consequently, lineages with a greater level of Phe and Tyr were expected to have a greater amount of biomass. Our results were consistent with the expectation, in which both transgenic Arabidopsis and CWRH lineages overexpressing the *EPSPS* gene had a significantly higher amount (*p* < 0.001) of fresh weight and dry weight than their corresponding non-transgenic counterparts normally expressing the *EPSPS* gene (Figure 7A–D).

For the 40-day-old Arabidopsis plants (*n* = 5 × 6), the increased ratios of the fresh weight and dry weight were about 22.9~24.5% and 20.3~27.4%, respectively, in the two transgenic lineages, compared with those in their corresponding non-transgenic counterparts (Figure 7A,B). However, for the 45-day-old CWRH plants (*n* = 5 × 6), the increased ratios of the fresh weight and dry weight were about 66.0~133.3% and 71.9~115.6%, respectively, in the two transgenic lineages, compared with those in corresponding non-transgenic counterparts (Figure 7C,D). Altogether, these results indicated that overexpression of the *EPSPS* gene substantially increased the biomass, both in dicot Arabidopsis and grass (CWRH) plants. Noticeably, the ratios of the increased biomass were much more prominent in the transgenic grass plants overexpressing the *EPSPS* gene than those in the transgenic Arabidopsis plants also overexpressing the *EPSPS* gene (Figure 7A–D).

## 4. Discussion

### 4.1. Increased Phe and Tyr Content by Overexpressing the EPSPS Transgene in Arabidopsis and CWRH Plants

Results obtained from this study demonstrated the substantially increased content of phenylalanine (Phe) and tyrosine (Tyr) both in transgenic dicot Arabidopsis and grass transgenic crop-weedy rice hybrid (CWRH) lineages. However, a great variation was observed in the increased content between all the transgenic lineages. The findings were based on a detailed comparison between transgenic plants (Arabidopsis and CWRH) that overexpressed the EPSPS transgene and their corresponding non-transgenic counterparts, who only normally expressed their endogenous EPSPS gene. The substantial increases in Phe and Tyr in transgenic plants are most likely due to overexpression of the *EPSPS* gene because previous studies also suggested that the content of the aromatic amino acids, including Phe and Tyr, can be regulated by the *EPSPS*-gene-related compounds [21,27,28]. Thus, the significantly increased contents of Phe and Tyr in the transgenic plants overexpressing the *EPSPS* gene provide a basis for studying the efficiency of applying Phe and Tyr to synthesize lignin and to produce biomass in dicot Arabidopsis having the PAL pathway and grass species having the bifunctional PTAL pathway.

Noticeably, a slightly greater proportion (ratio) of increased Tyr content than Phe was detected in the transgenic Arabidopsis and CWRH lineages overexpressing the *EPSPS* transgene. This phenomenon is probably because Tyr cannot be directly deaminated to synthesize lignin and biomass, although Tyr can be used by many other downstream pathways [15,29]. It is well known that Tyr cannot be directly deaminated to synthesize lignin by the dicot plants, such as Arabidopsis, which only has the monofunctional PAL pathway [11,29]. As reported by Schenck et al., Tyr serves as the precursor for many metabolites responsible for defenses to biotic stresses, pollinator attraction, and electron transport [15]. Thus, the results of this study have perfectly answered our first question in the Introduction, concerning the increased Phe and Tyr content associated with the overexpression of the *EPSPS* gene.

### 4.2. More Efficient Lignin and Biomass Production by Increased Phe and Tyr Content in CWRH Lineages 

Results from this study also clearly demonstrated substantially increased contents of total lignin and its three major monolignols (H-, S-, and G-lignin units) both in the transgenic Arabidopsis and CWRH lineages overexpressing the *EPSPS* gene, compared to their non-transgenic counterparts. The increased contents of the total lignin and the monolignols were most likely the consequence of significantly increased Phe and Tyr levels. Therefore, the increased ratios of Phe and Tyr from the non-transgenic to the transgenic Arabidopsis and CWRH lineages provide an ideal experimental system to accurately assess the efficiency of lignin biosynthesis and biomass production between the dicot and grass plants. Evidently, our results confirmed that the *EPSPS* gene expression-associated production of Phe and Tyr could determine the content of the key precursors for lignin biosynthesis and production of biomass both in dicot plants (e.g., Arabidopsis) only possessing the PAL pathway, as well as in the grass plants (e.g., CWRH) possessing both the PAL/PTAL pathways. In other words, the comparable experimental system, including non-transgenic lineages with a low level of Phe/Tyr and transgenic lineages with a high level of Phe/Tyr makes the assessment of lignin biosynthesis efficiency possible under the similar condition of increased precursor content in dicot and monocot plants.

In general, the proportion of increased total lignin (up to ~112%) and its H-, G-, and S-lignin units (up to ~109%, 106%, and 107%, respectively) was much greater in the transgenic grass CWRH lineages than that in the transgenic dicot Arabidopsis lineages (total lignin ~33%; H-, G-, and S-lignin units ~48%, 45%, and 60%), compared with their non-transgenic counterparts. This comparison was made under the condition that the proportion of increased Phe and Tyr contents was greater in the transgenic Arabidopsis lineages. Therefore, we concluded, based on the above findings, that lignin biosynthesis is much more efficient in grass plants possessing the bifunctional PTAL pathway than in dicots (dicotyledons) and probably in many other monocots (monocotyledons) only possessing the monofunctional PAL pathway. These findings address our third question concerning the increased efficiency of lignin biosynthesis in grass plants possessing the bifunctional PTAL pathway.

The increased efficiency of lignin biosynthesis in grass plants can be explained as follows. Grass species with PTAL can utilize both Phe and Tyr as precursors for lignin biosynthesis at the initial stage, which has much more abundant sources. In contrast, dicots and other plants with PAL can only use Phe, but not Tyr, for lignin biosynthesis, even though abundant Tyr is available. While grass plants can deaminate Tyr in the PTAL pathway to directly synthesize *p*-coumaric acid (Figure 1), a key step for lignin biosynthesis, along with the deamination of Phe in the PAL pathway to synthesize cinnamic acid (see details in Figure 1A,B). Whether the combination of abundant precursors and the “shortcut” of lignin biosynthesis further increases the efficiency in grass plants requires further investigation.

Our results further demonstrate substantially increased biomass production, as represented by increased fresh and dry weight, in transgenic Arabidopsis and CWRH lineages overexpressing the *EPSPS* gene compared to their non-transgenic counterparts. The significantly increased biomass is most likely a result of the considerable increases in Phe and Tyr contents due to the overexpressed *EPSPS* gene in the transgenic lineages. This is because Phe and Tyr serve as key precursors in the biosynthesis of lignin, which constitutes ~30% of dry matter (biomass) in plants [30,31]. Interestingly, the proportion of the increased fresh and dry weight of biomass was much more pronounced (up to ~133% and 116%) in the transgenic CWRH lineages, compared with the proportion (up to ~24% and 27%) in the transgenic Arabidopsis lineages. This finding strongly demonstrates that grass plants with the PTAL pathway can utilize both Phe and Tyr more efficiently to produce relevant secondary metabolites than dicot plants with the PAL pathway, which can only utilize Phe.

### 4.3. Implications of EPSPS Gene Overexpression in Carbon Fixation and Biofuel Production of Plants 

Results from the present study evidently demonstrate that overexpression of the exogenous *EPSPS* transgene through genetic engineering significantly enhances the biosynthesis of total lignin and the production of biomass in plants. In other words, genetic engineering mediates overexpression of the *EPSPS* transgene in plants, which can consume more carbon (CO_2_) in the atmosphere to synthesize more lignin and produce more biomass in plants, particularly in the grass species. Hence, plant species, particularly the grass species with both the bifunctional PTAL pathway and overexpression of the *EPSPS* gene, can serve as an important carbon sink because of the increased efficiency in deaminating Phe and Tyr for lignin and biomass production. Our findings have important implications for carbon fixation and biofuel production. 

Carbon dioxide (CO_2_) is the most important carbon source for photosynthesis in plants. Our results indicated significantly increased lignin biosynthesis in plants, particularly the grass species, overexpressing the *EPSPS* gene. Given that lignin biosynthesis consumes a lot of CO_2_ and that lignin is stored in plants as a part of biomass, being a huge carbon sink, we propose that the increased efficiency in synthesizing lignin and biomass by overexpressing the endogenous and exogenous *EPSPS* genes can largely promote carbon neutrality. A previous study reported greater increases in biomass content (~28%) in *EPSPS* transgenic Arabidopsis lineages than in their non-transgenic counterparts [20]. A recent report indicated that Arabidopsis and CWRH plants overexpressing the *EPSPS* gene could substantially promote carbon fixation due to the significant increases in CO_2_ consumption [32]. Altogether, these findings solidly support our proposal about the increased efficiency of consuming and fixing CO_2_ through increased dry-matter biosynthesis.

In addition, the substantially increased efficiency of lignin and biomass production by overexpressing the *EPSPS* gene can also be applied to biofuel production in plants. Many studies explored the prospects of applying lignin and biomass as a feedstock potential for valuable fuels and chemicals, expanding bioenergy opportunities [30,31,33]. Advanced knowledge of lignocellulosic biomass, which consists of cellulose, lignin, and hemicellulose, provides a promising source of raw materials for biofuels and lignin-derived fuels [33]. Advanced technologies will allow us to reduce our dependency on fossil fuels and greenhouse gas emissions in the future. Therefore, the application of genetic engineering to regulate the expression of the *EPSPS* gene in the shikimate pathway of plants will play important roles in carbon fixation and biofuel production. 

## 5. Conclusions

In this study, we found substantially increased phenylalanine (Phe) and tyrosine (Tyr) in the transgenic Arabidopsis and CWRH plants overexpressing the exogenous *EPSPS* transgene, compared with their non-transgenic counterparts only normally expressing the endogenous *EPSPS* gene. The grass (CWRH) plants possessing the bifunctional PTAL pathway exhibited greater efficiency in lignin biosynthesis (increased by 74.0~111.6%) and biomass production (increased by 71.9~115.6% in dry matter) compared to the dicot (Arabidopsis) plants, although the increased Phe and Tyr levels were comparable. Obviously, the grass species that have evolved the bifunctional PTAL pathway become more efficient in using both Phe and Tyr as precursors for lignin biosynthesis. These findings have significant implications in biomass production, carbon fixation, and bioenergy exploration in plants through enhanced efficiency of lignin biosynthesis by employing genetic engineering biotechnologies to manipulate the relevant genes in the phenylpropanoid metabolic pathways. The availability of such knowledge and technologies will facilitate the implementation of our goals through enhanced biosynthesis efficiency for phenylpropanoid-pathway-associated metabolites.

## Data Availability

All the data was shown in the article.

## References

[1] Weng J.-K., Chapple C. (2010). The Origin and Evolution of Lignin Biosynthesis. New Phytol..

[2] Boerjan W., Ralph J., Baucher M. (2003). Lignin biosynthesis. Annu. Rev. Plant Biol..

[3] Liu Q., Luo L., Zheng L. (2018). Lignins: Biosynthesis and Biological Functions in Plants. Int. J. Mol. Sci..

[4] Kenrick P., Crane P.R. (1997). The origin and early evolution of plants on land. Nature.

[5] Martone P.T., Estevez J.M., Lu F., Ruel K., Denny M.W., Somerville C., Ralph J. (2009). Discovery of lignin in seaweed reveals convergent evolution of cell-wall architecture. Curr. Biol..

[6] Xie M., Zhang J., Tschaplinski T.J., Tuskan G.A., Chen J.-G., Muchero W. (2018). Regulation of Lignin Biosynthesis and Its Role in Growth-Defense Tradeoffs. Front. Plant Sci..

[7] Gou M., Yang X., Zhao Y., Ran X., Song Y., Liu C.-J. (2019). Cytochrome *b*_5_ Is an Obligate Electron Shuttle Protein for Syringyl Lignin Biosynthesis in Arabidopsis. Plant Cell.

[8] Bonawitz N.D., Kim J.I., Tobimatsu Y., Ciesielski P.N., Anderson N.A., Ximenes E., Maeda J., Ralph J., Donohoe B.S., Ladisch M. (2014). Disruption of Mediator rescues the stunted growth of a lignin-deficient *Arabidopsis* mutant. Nature.

[9] Maeda H., Dudareva N. (2012). The Shikimate Pathway and Aromatic Amino Acid Biosynthesis in Plants. Annu. Rev. Plant Biol..

[10] Feduraev P., Skrypnik L., Riabova A., Pungin A., Tokupova E., Maslennikov P., Chupakhina G. (2020). Phenylalanine and Tyrosine as Exogenous Precursors of Wheat (*Triticum aestivum* L.) Secondary Metabolism through PAL-Associated Pathways. Plants.

[11] Barros J., Serrani-Yarce J.C., Chen F., Baxter D., Venables B.J., Dixon R.A. (2016). Role of bifunctional ammonia-lyase in grass cell wall biosynthesis. Nat. Plants.

[12] Barros J., Dixon R.A. (2020). Plant Phenylalanine/Tyrosine Ammonia-lyases. Trends Plant Sci..

[13] Vanholme R., Meester B.D., Ralph J., Boerjan W. (2019). Lignin biosynthesis and its integration into metabolism. Curr. Opin. Biotechnol..

[14] Kumar A.K., Parikh B.S., Pravakar M. (2016). Natural deep eutectic solvent mediated pretreatment of rice straw: Bioanalytical characterization of lignin extract and enzymatic hydrolysis of pretreated biomass residue. Environ. Sci. Pollut. Res. Int..

[15] Schenck C.A., Maeda H.A. (2018). Tyrosine biosynthesis, metabolism, and catabolism in plants. Phytochemistry.

[16] Melero J.A., Iglesias J., Garcia A. (2012). Biomass as renewable feedstock in standard refinery units. Feasibility, opportunities and challenges. Energy Environ. Sci..

[17] Mood S.H., Golfeshan A.H., Tabatabaei M., Jouzani G.S., Najafi G.H., Gholami M., Ardjmand M. (2013). Lignocellulosic biomass to bioethanol, a comprehensive review with a focus on pretreatment. Renew. Sustain. Energy Rev..

[18] Wang K., Wang Y., Wen H., Zhang X., Yu J., Wang Q., Han S., Wang W. (2023). Biomass carbon sink stability of conifer and broadleaf boreal forests: Differently associated with plant diversity and mycorrhizal symbionts?. Environ. Sci. Pollut. Res..

[19] Wang W., Xia H., Yang X., Xu T., Si H.J., Cai X.X., Wang F., Su J., Snow A.A., Lu B.-R. (2014). A novel 5-enolpyruvoylshikimate-3-phosphate (EPSP) synthase transgene for glyphosate resistance stimulates growth and fecundity in weedy rice (*Oryza sativa*) without herbicide. New Phytol..

[20] Fang J., Nan P., Gu Z., Ge X., Feng Y.-Q., Lu B.-R. (2018). Overexpressing Exogenous 5-Enolpyruvylshikimate-3-Phosphate Synthase (EPSPS) Genes Increases Fecundity and Auxin Content of Transgenic Arabidopsis Plants. Front. Plant Sci..

[21] Wu J., Fang J., Cai X.X., Lu B.-R. (2020). Overexpressing 5-enolpyruvylshikimate-3-phosphate Synthase Gene Increases Lignin Content of Transgenic Progeny Derived from Hybrids of *EPSPS* Transgenic Rice with Weedy and Wild Rice. J. Fudan Univ. (Nat. Sci.).

[22] Jiang X.-Q., Yang X., Lu B.-R. (2021). Increased Longevity and Dormancy of Soil-Buried Seeds from Advanced Crop–Wild Rice Hybrids Overexpressing the *EPSPS* Transgene. Biology.

[23] Ma T., Yuan Y., Lin K., Nan P., Lu B. (2020). Overexpression of *EPSPS* Transgene Enhances Chlorophyll Synthesis in Hybrid Progeny between Cultivated Rice and Weedy Rice. J. Fudan Univ. (Nat. Sci.).

[24] Livak K.J., Schmittgen T.D. (2001). Analysis of Relative Gene Expression Data Using Real-Time Quantitative PCR and the 2^−ΔΔCT^ Method. Methods.

[25] Liu Z., Rochfort S. (2013). A fast liquid chromatography–mass spectrometry (LC–MS) method for quantification of major polar metabolites in plants. J. Chromatogr. B.

[26] Song Y., Wu Y.-C., Zhang Y., Wang Z.-Z. (2011). Determination of Lignin Content and Lignin Monomer Composition in *Salvia miltiorrhiza* Bge. J. Anal. Sci..

[27] Vila-Aiub M.M., Yu Q., Powles S.B. (2019). Do plants pay a fitness cost to be resistant to glyphosate?. New Phytol..

[28] Hildebrandt T.M., Nesi A.N., Araújo W.L., Braun H.-P. (2015). Amino Acid Catabolism in Plants. Mol. Plant.

[29] Cochrane F.C., Davin L.B., Lewis N.G. (2004). The *Arabidopsis* phenylalanine ammonia lyase gene family: Kinetic characterization of the four PAL isoforms. Phytochemistry.

[30] Cao L., Zhang C., Chen H., Tsang D.C.W., Luo G., Zhang S., Chen J. (2017). Hydrothermal liquefaction of agricultural and forestry wastes: State-of-the-art review and future prospects. Bioresour. Technol..

[31] Zhu D., Zhang P., Xie C., Zhang W., Sun J., Qian W.-J., Yang B. (2017). Biodegradation of alkaline lignin by *Bacillus ligniniphilus* L1. Biotechnol. Biofuels.

[32] Sun L.-X., Li N., Yuan Y., Wang Y., Lu B.-R. (2024). Reduced Carbon Dioxide by Overexpressing *EPSPS* Transgene in Arabidopsis and Rice: Implications in Carbon Neutrality through Genetically Engineered Plants. Biology.

[33] Kocaturk E., Salan T., Ozcelik O., Alma M.H., Candan Z. (2023). Recent Advances in Lignin-Based Biofuel Production. Energies.

